# Cytostasis and morphological changes induced by mifepristone in human metastatic cancer cells involve cytoskeletal filamentous actin reorganization and impairment of cell adhesion dynamics

**DOI:** 10.1186/1471-2407-13-35

**Published:** 2013-01-26

**Authors:** BreeAnn N Brandhagen, Chelsea R Tieszen, Tara M Ulmer, Maria S Tracy, Alicia A Goyeneche, Carlos M Telleria

**Affiliations:** 1 Division of Basic Biomedical Science, Sanford School of Medicine of The University of South Dakota, 414 East Clark Street, Vermillion, SD, 57069, USA

## Abstract

**Background:**

Changes in cell shape and plasticity in cytoskeletal dynamics are critically involved in cell adhesion, migration, invasion and the overall process of metastasis. Previous work in our laboratory demonstrated that the synthetic steroid mifepristone inhibited the growth of highly metastatic cancer cells, while simultaneously causing striking changes in cellular morphology. Here we assessed whether such morphological alterations developed in response to cytostatic concentrations of mifepristone are reversible or permanent, involve rearrangement of cytoskeletal proteins, and/or affect the adhesive capacity of the cells.

**Methods:**

Cancer cell lines of the ovary (SKOV-3), breast (MDA-MB-231), prostate (LNCaP), and nervous system (U87MG) were exposed to cytostatic concentrations of mifepristone and studied by phase-contrast microscopy. The transient or permanent nature of the cytostasis and morphological changes caused by mifepristone was assessed, as well as the rearrangement of cytoskeletal proteins. De-adhesion and adhesion assays were utilized to determine if mifepristone-arrested and morphologically dysregulated cells had abnormal de-adhesion/adhesion dynamics when compared to vehicle-treated controls.

**Results:**

Mifepristone-treated cells displayed a long, thin, spindle-like shape with boundaries resembling those of loosely adhered cells. Growth arrest and morphology changes caused by mifepristone were reversible in SKOV-3, MDA-MB-231 and U87MG, but not in LNCaP cells that instead became senescent. All cancer cell types exposed to mifepristone displayed greatly increased actin ruffling in association with accelerated de-adhesion from the culture plate, and delayed adhesion capacity to various extracellular matrix components.

**Conclusions:**

Cytostatic concentrations of mifepristone induced alterations in the cellular structure of a panel of aggressive, highly metastatic cancer cells of different tissues of origin. Such changes were associated with re-distribution of actin fibers that mainly form non-adhesive membrane ruffles, leading to dysregulated cellular adhesion capacity.

## Background

Originally developed as an anti-glucocorticoid agent in the 1980s, the synthetic steroid mifepristone was also found to modulate the progesterone receptor. This unexpected finding led mifepristone to be rapidly repurposed for its use for early termination of pregnancy. However, aside from this most common usage, mifepristone has been proven effective as a growth inhibitor in endometriosis [1,2], uterine fibroids [3-5], and benign cases of meningioma [6]. In relation to cancer cell growth, mifepristone was shown to have antiproliferative effects in cervical [7], breast (reviewed in [8]), endometrial [9-12], ovarian [13-17], gastric [18] and prostate cancer cells [19,20]. In mice with spontaneous lung cancer or leukemia, mifepristone improved quality of life and longevity [21,22]. Also, mifepristone given daily to case-study patients with widely metastatic thymic, renal, colon, or pancreatic cancers no longer responding to chemotherapy significantly improved patient quality of life [23]. As early as 1998, the suggestion of the use of mifepristone as a therapeutic option for highly aggressive, metastatic cancers was introduced [24]. However, since then there has been little investigation pursued in this subject area.

Previous work in our laboratory demonstrated that mifepristone: i) arrests the growth of ovarian cancer cells by inhibiting DNA synthesis and halting progression of the cell cycle at the G_1_-S transition [17]; ii) prevents repopulation of remnant ovarian cancer cells when added after platinum or platinum/taxane therapies [15,25]; and iii) has growth inhibitory effects on various cell types representing aggressive cancers of the prostate, breast, nervous system, and bone [26]. Of particular interest in this previous study [26] was the observation that the cells were not only growth inhibited in response to mifepristone, but that they also displayed major changes in their morphological features.

Changes in cellular structure are a consequence of the rearrangement of cytoskeletal proteins, and are critically involved in adhesion turnover and polarized cell migration required for the success of the metastatic process [27,28]. In this work we studied whether mifepristone-induced variations in morphology, while cells undergo cytostasis, are dependent on the continuous presence of the drug, and whether there is an association between cytostasis, redistribution of filamentous actin (F-actin) and tubulin filaments, and altered adhesion capacity to extracellular matrix proteins. We report that mifepristone-induced cytostasis and morphological changes were comparable across a panel of different cancer cell lines, with cells developing a thin cytoplasm with neurite-like protrusions. Mifepristone also impacted the distribution of cytoskeletal actin fibers, with increased concentrations in membrane ruffles, and of tubulin filaments mainly allocating to the neurite-like cellular extensions. These observations were associated with an overall impairment in the dynamics of the adhesive capacity of the cells manifested by accelerated detachment when the drug was applied to adherent cells, and impaired attachment of cells that were pre-treated with the drug and then allowed to adhere to extracellular matrix proteins in drug-free media. These results provide evidence supporting a potential role of mifepristone in altering the metastatic capacity of cancer cells.

## Methods

### Cell culture and *in vitro* exposure to mifepristone

The human ovarian carcinoma cell line SKOV-3, the human breast carcinoma cell line MDA-MB-231, the human glioblastoma cell line U87MG, and the human prostate carcinoma cell line LNCaP were obtained from the American Type Culture Collection (ATCC, Manassas, VA) and cultured as previously detailed [26]. Treatment of the cells with mifepristone (Sigma Chemical Company, St. Louis, MO) used a 20,000 μM stock solution of the drug in DMSO (Mediatech, Herndon, VA). The maximal concentration of DMSO in medium was 0.15% (v/v). The final working concentrations of mifepristone were 23.5 μM for SKOV-3 cells, 30 μM for MDA-MB-231 cells, 20 μM for U87MG cells, and 20 μM for LNCaP cells. All cells were cultured at 37°C in a humidified atmosphere in the presence of 5% CO_2_. The human fibroblast cell line WI-38 used as negative control of cell senescence was obtained from ATCC and was maintained in DMEM (Mediatech) supplemented with 10% FBS (Atlanta Biologicals, Lawrenceville, GA), 10 mM HEPES, 4 mM L-glutamine (Sigma), 1 mM sodium pyruvate (Mediatech), 100 IU penicillin (Mediatech), and 100 μg/ml streptomycin (Mediatech). All cells were cultured at 37°C in a humidified atmosphere in the presence of 5% CO_2_.

### Time-course of morphology

Cells were seeded in 6-well plates at a density of 100,000 cells per well and allowed 24 h for attachment. Using previously established cytostatic doses of mifepristone (SKOV-3: 23.5 μM, MDA-MB-231: 30 μM, U87MG: 20 μM, LNCaP: 20 μM) [26]), treatment was performed for 72 h, during which morphology changes were assessed by phase contrast microscopy. Images of vehicle and mifepristone-treated cells were taken every 12 h throughout the experimental period using a Zeiss Axiovert M200 inverted microscope with a phase contrast objective (Carl Zeiss, Thornwood, NY). Additionally, SKOV-3 cells were grown in chamber slides at a concentration of 10,000 cells per well and subjected or not to treatment with mifepristone. At the end of incubation, the cells were fixed with 4% paraformaldehyde (Sigma) and stained with hematoxylin (Sigma).

### Reversal of cell proliferation and morphology

In order to determine the long-term effect of mifepristone treatment, cell morphology was assessed after removal of treatment media. After 72 h of treatment, mifepristone-containing media was removed, and media for all cells was replaced with control media. Phase contrast images were taken at 0 h, 12 h, 24 h, 48 h, and 72 h after mifepristone withdrawal to observe cell morphology. In addition to images taken at each time-point, cell number and viability were determined using the Guava EasyCyte Mini microcapillary cytometer (Guava Technologies, Hayward, CA). Samples were collected at the beginning of the experiment, for vehicle and mifepristone-treated cells after 72h of treatment, and at each time-point after treatment withdrawal. Triplicate wells were trypsinized, the cells pelleted by centrifugation at 500 *g* for 5 minutes, and resuspended in an appropriate volume of PBS. A 1:10 (v/v) dilution of cell suspension and ViaCount reagent (Guava Technologies) was prepared for each sample. The data were acquired and analyzed using the CytoSoft 4.1 software (Guava Technologies).

### Senescence Associated (SA)-β-galactosidase staining

Using 6-well plates, LNCaP cells were plated at a density of 50,000 cells per well. Cells were treated with mifepristone for 72 h followed by vehicle for 5 days, or vehicle, 5% charcoal-stripped (CS)-FBS, or 10% CS-FBS media for 8 days prior to SA-β-galactosidase staining. Cells were fixed in 0.5% glutaraldehyde at 4°C for 10 min and then washed three times with PBS. Cells were then incubated for 3 h with 5-bromo-4-chloro-3-indolyl-beta-D-galactopyranoside (X-gal) staining solution consisting of 1 mg/ml X-gal, 40 mM citric acid/sodium phosphate (pH 6.0), 5 mM potassium ferricyanide, 5 mM potassium ferrocyanide, 150 mM NaCl, and 2 mM MgCl_2_. Following incubation, cells were washed briefly with PBS and stored in 100% methanol for analysis and imaging. Cells that expressed SA-β-galactosidase were stained blue when viewed using a Zeiss Axiovert M1-Imager (Carl Zeiss) microscope. Senescence was quantified as the number of blue-stained cells per field and expressed as a percent of total number of cells per field and corrected against a negative control. The average of 9 fields per well was calculated, with 3 wells per treatment group. Positive controls for senescence staining were LNCaP cells that had been depleted of steroids by culture in 5% or 10% CS-FBS media as reported [29]. The negative control for senescence was a culture of WI-38 fibroblasts maintained in FBS-containing media.

### Immunocytochemistry

All cell lines were plated in 8-well chamber slides at a density of 5,000 cells per well. Cells were allowed to attach for 24 h before treatment began. Wells were treated with vehicle or media containing mifepristone for 72 h at doses tailored to induce cytostasis and morphological changes to individual cell lines. Following treatment cells were fixed according to Waterman-Storer et al. [30] to ensure stabilization of microtubules. First, cells were prefixed for 5 min in a solution of 1% paraformaldehyde, 0.5% Triton X-100, prepared in PHEM buffer [60 mM Na PIPES, 25mM Hepes, 10 mM EGTA, 4 mM MgSO_4_, pH 7.2]. Next, cells were fixed for 15 min in a solution of 1% paraformaldehyde, 0.5% glutaraldehyde, prepared in PHEM. This was followed by 3 washes with PHEM buffer alone. Finally, any free aldehydes were blocked by 3 × 5 min incubations with 1 mg/ml sodium borohydride. Cells were rinsed with PBS multiple times and stored in PBS at 4°C until staining. Following fixation, cells were incubated with a blocking buffer [PBS, 5% normal goat serum (NGS), 0.1% Triton X-100] for 20 min at room temperature. This was followed by 1 h incubation with 1 μg/ml of anti-bovine α-tubulin, mouse monoclonal antibody (A-11126, Molecular Probes, Eugene, OR). Any unbound antibody was removed by 3 x 5 min washes with washing buffer (PBS, 0.1% Triton X-100). Cells were then incubated at room temperature for 1 h with 1 μg/ml of Alexa Fluor^®^ 488 Goat Anti-Mouse IgG (H + L) (A-11001, Molecular Probes). From this point onward, cells were protected from light. The unbound secondary antibody was removed with 3 x 5 min washes with washing buffer. To access F-actin distribution, cells were incubated with Alexa Fluor^®^ 594 phalloidin (Invitrogen, Grand Island, NY). A 6.6 μM stock solution of Alexa Fluor^®^ 594 phalloidin was diluted with PBS containing 1% BSA in a 1:40 ratio. Cells were incubated with the phalloidin staining solution for 20 min. Finally, cells were washed multiple times with PBS and mounted using Vectashield^®^ Hard-Set™ Mounting Medium with DAPI (Vector Laboratories Inc., Burlingame, CA). Cover slips were added and slides were allowed to set at room temperature for 15 min. Slides were then stored at 4°C, protected from light. Images were taken using a confocal Olympus FV1000 microscope with FluoView^®^ software.

### SDS-PAGE and western blotting

Cells were treated with vehicle or mifepristone for 72 h, after which cultures were trypsinized, stained with trypan blue, and counted using a hemacytometer. Equal numbers of viable vehicle and mifepristone-treated cells were then pelleted, washed twice with PBS, and snap frozen followed by storage at −80°C. Whole cell extracts were obtained, protein quantitated, separated by SDS-PAGE, electro-transferred to PVDF membranes, and then probed for 1 h at room temperature using primary antibodies against α-tubulin (A-11126; 1:1,000; Molecular Probes), β-actin (clone AC-15; 1:10,000; Sigma), or GAPDH (ab94985; 1:8,000; Abcam Inc., Cambridge, MA).

### De-adhesion assay

Cells were first grown to 50% confluence in 6-well plates, and then treated with vehicle or mifepristone-containing media for 72 h. Following treatment, cells were exposed to 0.025% trypsin/0.265 mM EDTA for 30 sec, 2 min or 4 min. Detached cells were removed with a washing of PBS. Cells remaining adhered were fixed with 100% methanol, stained with crystal violet, and quantified using bright field microscopy. Cell adhesion was expressed as percent of adherent cells for each of the times of exposure relative to the adhesion measured in a culture not exposed to trypsin (considered to be 100%).

### Adhesion assays

The first adhesion assay was performed under sterile conditions using the CytoSelect 48-Well Cell Adhesion Assay (CBA-070, Cell Biolabs Inc., San Diego, CA). Briefly, the adhesion plates containing various extracellular matrix components (fibronectin, collagen I, collagen IV, fibrinogen, or laminin) were allowed to warm up at room temperature for 10 min. Cell suspensions were then prepared containing 1 × 10^6^ cells per ml in serum-free media with or without mifepristone; 150 μl of each cell suspension was added to the appropriate wells and the plates were incubated for 60 min at 37°C in a humidified atmosphere in the presence of 5% CO_2_. The media was discarded from each well and all wells were washed 4–5 times with 250 μl PBS. PBS was removed and 200 μl of the provided cell stain solution was added to each well. The plates were incubated for 10 min at room temperature. After incubation, the cell stain solution was removed and each well was washed 4–5 times with 500 μl of deionized water. The final wash was discarded and wells were allowed to air dry. Next, 200 μl of provided extraction solution was added to each well and the plates were incubated for 10 min on an orbital shaker at room temperature. Finally, 150 μl of each sample was transferred to 96-well microtiter plates and the optical density at 540 nm was measured using a Titertek Multiskan MCC/340 Microplate Reader II (Dupont, Labsystems, Finland).

When adhesion to fibronectin was further studied, cells were cultured in the presence of 20 μM mifepristone for 72 h or left untreated in controls. The cells were trypsinized and incubated in suspension for 20 min to allow recovery from trypsinization. Thereafter, 100,000 cells were placed per 35 mm diameter plates that had been pre-coated with 0.1% fibronectin (Sigma), and were incubated for various times. Cells were fixed with methanol and stained with crystal violet. Counting was achieved under a microscope by recording number of adherent cells per 20 X microscopic field.

### Statistical analysis

Data processing and statistical analysis were performed using GraphPad Prism (GraphPad Prism Software, Inc., San Diego, CA). All data are represented as means ± SEM, and statistical significance was defined as *P* < 0.05. To compare among experimental groups, one-way ANOVA followed by the Tukey’s multiple comparison test or two-way ANOVA followed by the Bonferroni’s multiple comparison test were used as appropriate. To study significant differences between two groups, the Student’s *t*-test was used.

## Results

### Cytostatic concentrations of mifepristone cause morphological changes in cancer cell lines of the ovary, breast, prostate, and nervous system

**Figure 1:**
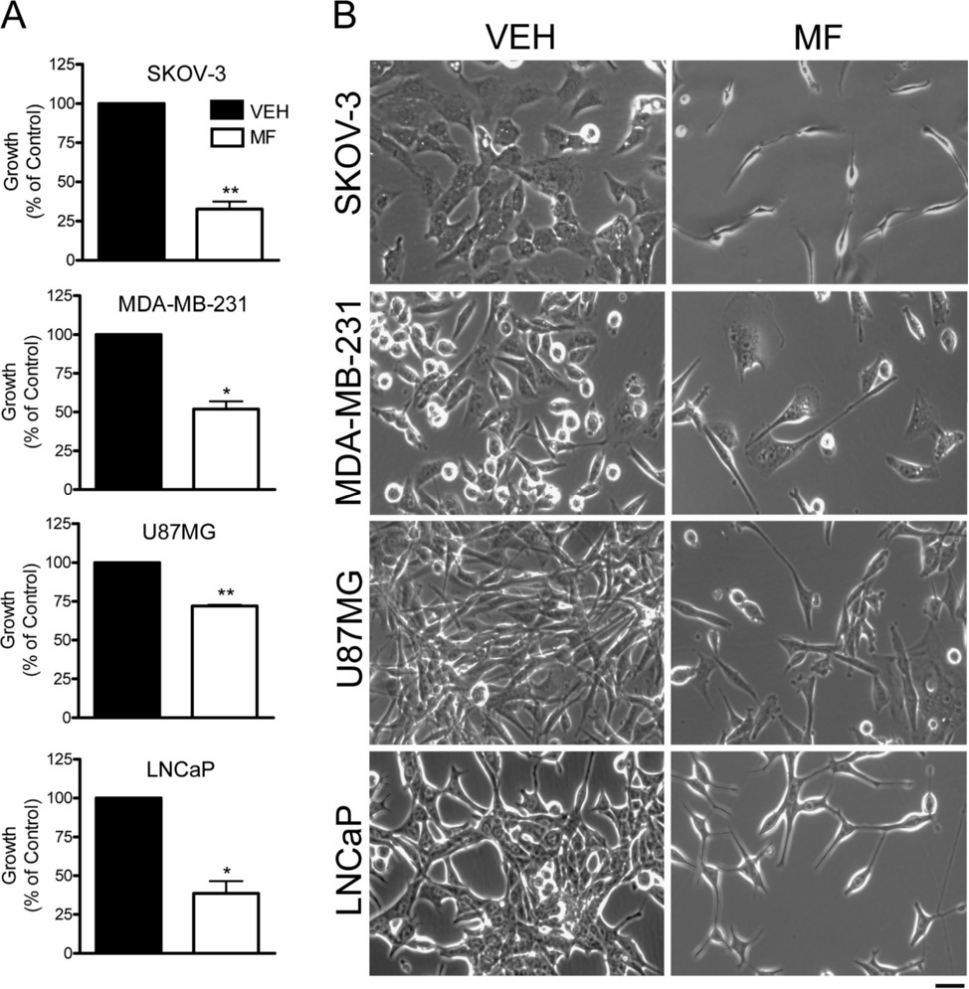
**Cells lines representing cancers of the ovary (SKOV-3), breast (MDA-MB-231), nervous system (U87MG), and prostate (LNCaP) display distinct morphological changes in response to mifepristone treatment.** Equal number of cells were plated and allowed to attach for 24 h. Cells were then exposed to vehicle (VEH)-containing media, or media containing a previously tailored cytostatic dose of mifepristone (MF) for a period of 72 h. At the end of the experiment the total number of cells was recorded by microcytometric analysis **(A)** and images were taken using phase contrast microscopy **(B)**. Cells were exposed to the following cytostatic concentrations of MF: 23.5 μM for SKOV-3, 30 μM for MDA-MB-231, and 20 μM for U87MG and LNCaP. *p < 0.01 and **p < 0.005 vs. VEH (Student’s *t*-test). Scale bar = 50 μm.

In a previous study we made the serendipitous observation that when cancer cells of various tissues of origin were exposed to concentrations of mifepristone that inhibited their growth [26], the cells displayed major changes in shape when compared to untreated growing cells. In the present work, highly aggressive cell lines representing cancers of the ovary, breast, prostate, and nervous system were selected for further analysis. Treatment with a previously tailored cytostatic dose of mifepristone for each cell line was administered for a period of 72 h, and images were taken using phase contrast microscopy every 12 h. At the end of the incubation period the number of cells was significantly reduced in mifepristone-treated wells when compared to their vehicle-treated counterparts (Figure 1A). The growth inhibitory effect of mifepristone was confirmed in microscopic images showing fewer cells present in mifepristone-treated cultures after 72 h incubation (Figure 1B). Phase contrast views across the panel of cell lines reveal that mifepristone-treated cells display a long, thin, spindle-like shape with boundaries resembling those of cells loosely adhered. The neurite-like extensions induced by mifepristone appear to reach out to other cells in the field (Figure 1B). The morphological changes induced by mifepristone began to be appreciated within 24–48 h of treatment (Additional file 1: Figure S1). Further details of thinning of the cytoplasm can be clearly appreciated upon hematoxylin staining in SKOV-3 cells that were exposed to 20 μM mifepristone for 72 h, when compared to vehicle-treated controls (Additional file 2: Figure S2). The morphological changes caused by mifepristone in all cell lines also occurred when cells were cultured at high density (results not shown). This confirmed the intrinsic effect of mifepristone on cell shape, and that observed morphological changes were not a result of perceived bias, owing to the reduced cellular densities commonly observed in cultures exposed to mifepristone as a consequence of the cytostatic effect of the steroid.

### Cellular proliferation and morphological changes are reversible upon removal of mifepristone in all cell lines except for LNCaP prostate cancer cells that undergo cellular senescence

**Figure 2:**
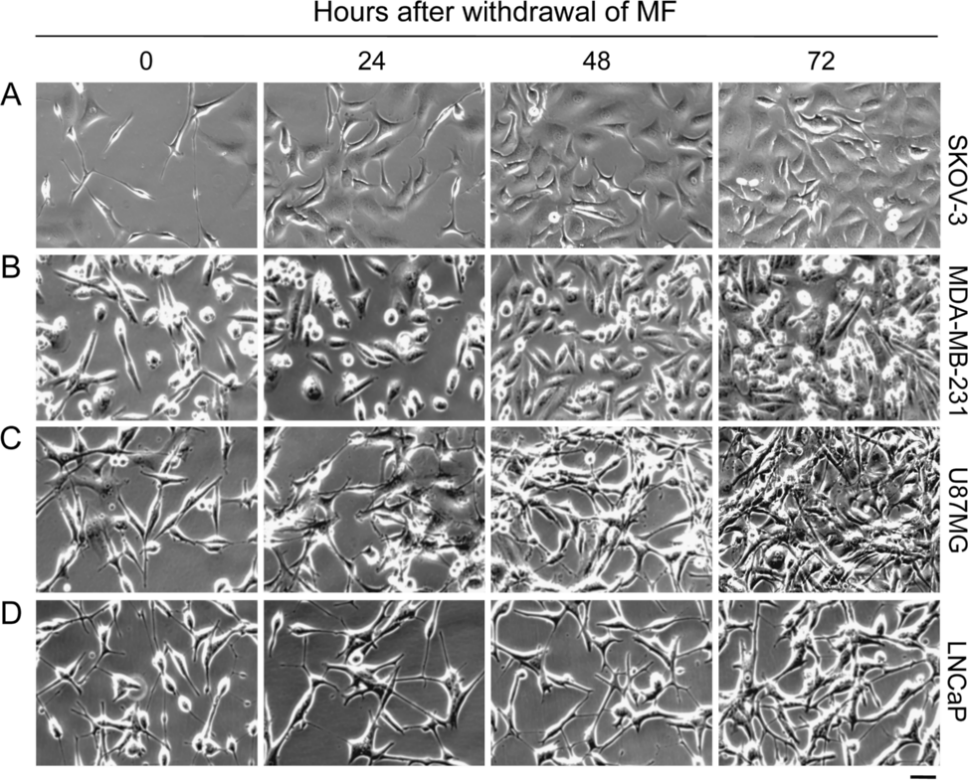
**Time-course effect of mifepristone withdrawal on cancer cells of the ovary (A), breast (B), prostate (C), and nervous system (D).** Cells were seeded at a density appropriate for exponential growth for each cell line, allowed to adhere for 24 h, and then exposed to the previously determined cytostatic concentrations of mifepristone for 72 h. Thereafter mifepristone-containing media was replaced with normal growth media and images were taken using phase contrast microscopy after 0, 24, 48 or 72 h **(A-D)**. Scale bar = 50 μm.

**Figure 3:**
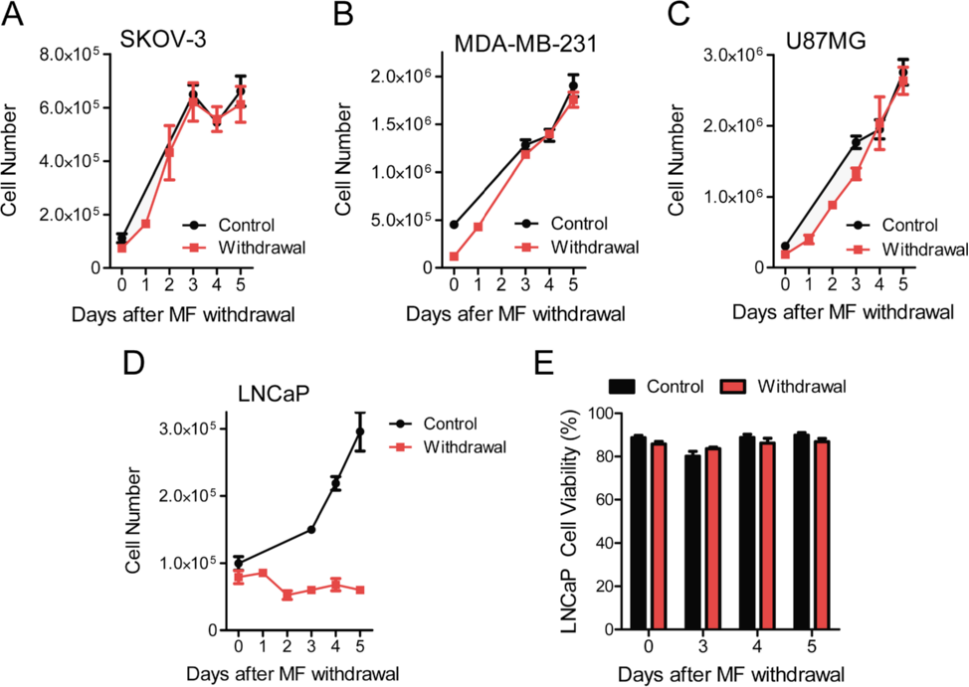
**Long-term effect of mifepristone on tumor cell lines of the ovary (A), breast (B), nervous system (C), and prostate (D, E).** Cells were seeded at a density appropriate for each cell line, allowed to adhere for 24 h, and then exposed to concentrations of mifepristone (MF) specific for each cell line for 72 h. Thereafter, triplicate wells were harvested by trypsinization and counted by microcytometry. Remaining wells were returned to vehicle treatment and monitored for 5 days, during which time triplicate wells were harvested and counted every 24 h. Growth expressed as number of cells per well are shown for each cell line **(A-D)**. Data points represent the mean ± s.e.m of 3 independent experiments completed in triplicate. Viability of LNCaP cells was also determined by microcytometric analysis **(E)**.

**Figure 4:**
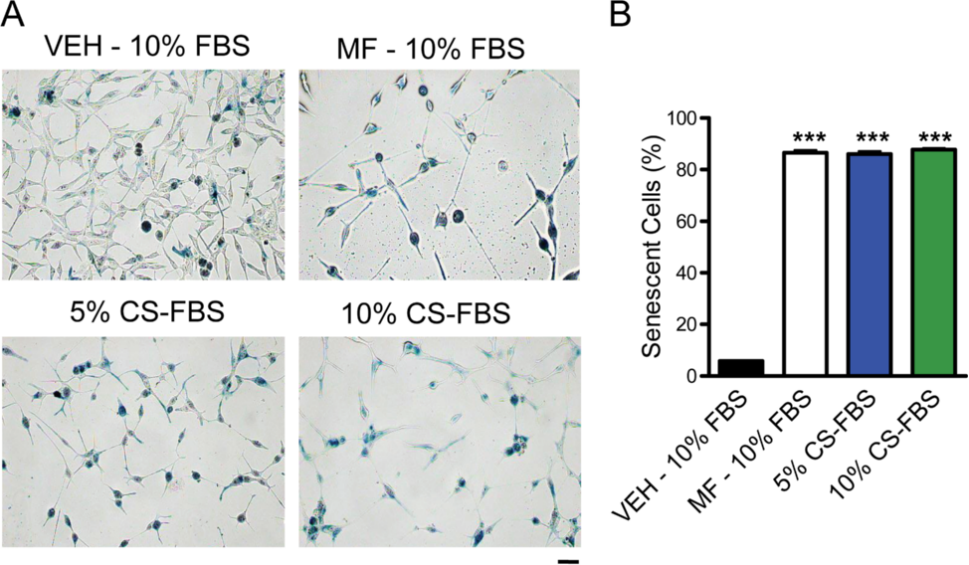
**Mifepristone treatment induces senescence in LNCaP cells.** LNCaP cells were seeded at 50,000 cells per well and treated with mifepristone (MF) for 72 h followed by vehicle (VEH) for 5 days; or vehicle, 5% or 10% charcoal-stripped (CS)-FBS-containing media for 8 days. SA-β-galactosidase staining was performed as surrogate marker of senescent cells. Relative senescence was quantified as the number of cells with blue cytoplasm per field and expressed as the percent of total number of cells per field **(A, B)**. Nine fields per well were quantified and completed in triplicate for each treatment group. All treatment groups were corrected against the background provided by the negative control, WI-38 cells that do not undergo senescence (data not shown). *** p < 0.0001 vs. VEH (one-way ANOVA followed by Tukey’s post-test). Scale bar = 50 μm.

To determine the long-term effect of mifepristone on cell proliferation and morphology, each cell line was cultured in the presence of vehicle or mifepristone for 72 h. Thereafter, mifepristone was removed from the cultures and replaced with media lacking the drug for 5 days. Subsequently, cultures were imaged and cell number was determined every 24 h to monitor for reversal of morphology and proliferation. While the proliferation of SKOV-3, MDA-MB-231, and U87MG cells remained relatively slow 1–2 days post-treatment, by day 3 after mifepristone withdrawal, cultures were proliferating at the same rate as cultures never treated with the synthetic steroid (Figure 2A-C and Figure 3A-C). Of note is that mifepristone pretreated cultures, when re-growing upon drug withdrawal, did not exceed the proliferation rate of their vehicle counterparts, but returned to a comparable doubling time (Figure 3A-C). In contrast, release of LNCaP cells from mifepristone treatment did not result in a return to normal proliferation (Figure 2D). Instead, mifepristone-pretreated LNCaP cultures failed to resume growth and remained with a steady state cell number through 5 days of normal culture conditions, while vehicle cultures increasingly proliferated (Figure 3D). Such regrowth was not observed either when extending the incubation in drug-free media for 9 days (Additional file 3: Figure S3). While LNCaP mifepristone-treated cultures did not resume normal proliferation, the cells did not show signs of lethality either, as indicated by morphological features (Figure 2D) and viability (Figure 3E) comparable to those of untreated cells. To determine whether the lack of growth of LNCaP cells upon removal of mifepristone is consequence of a permanent cell cycle arrest associated with cellular senescence, we stained mifepristone-pretreated LNCaP cells for SA-β-galactosidase activity. Results shown in Figure 4A reveal that while cultures not treated with mifepristone display low percentage of SA-β-galactosidase positive cells, such number remarkably increased in cultures under the presence of mifepristone. The increase in the percentage of SA-β-galactosidase positive cells induced by mifepristone was similar to that achieved when LNCaP cells were cultured in steroid-deprived medium, a condition reported to induce senescence in this cell line (Figure 4B) [29].

### Cytostatic doses of mifepristone dysregulate the cytoskeletal architecture of cancer cells

**Figure 5:**
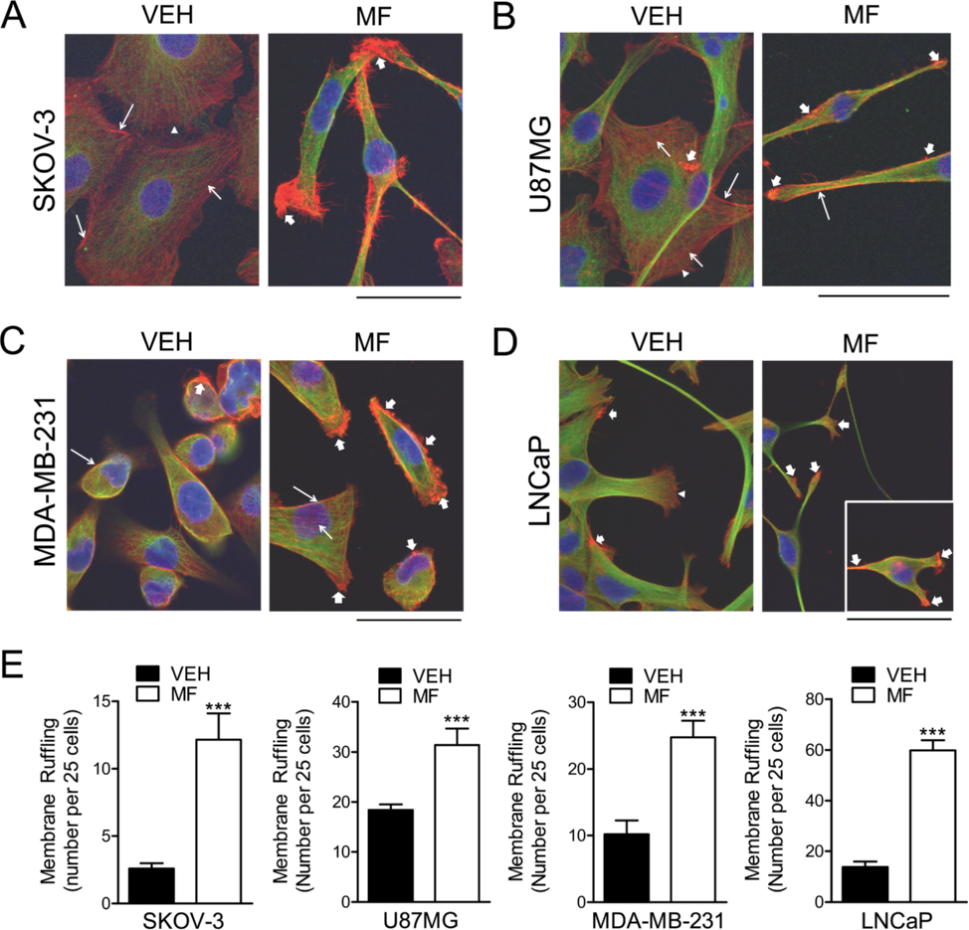
**Effect of mifepristone on cytoskeletal actin fibers and tubulin filaments.** SKOV-3 **(A)**, U87MG **(B)**, MDA-MB-231 **(C)** or LNCaP **(D)** cells were cultured in the presence of vehicle (VEH) or a cytostatic concentration of mifepristone (MF) for 72 h, following which immunofluorescence was used to visualize the cytoskeletal protein α-tubulin. AlexaFluor^®^ 594 phalloidin was utilized to visualize F-actin and DAPI to label cell nuclei. Images were taking using confocal microscopy. Scale bar = 50 μm. The inset in panel **D** represents a cell that was in a different field within the same image and that denotes the characteristic increase in membrane ruffles induced by MF (for the complete image see Additional file 4: Figure S4). In **A-D**, long, thin arrows, cortical actin; short, thin arrows, stress fibers; arrowheads, lamellipodia; short, wide arrows, membrane ruffles. Panel **E** represents the quantification of the membrane ruffles in culture for all cell lines studied in response to MF. Ruffles were counted from confocal microscopy images. We expressed membrane ruffling as number of ruffles counted every 25 cells, after assessing a minimum of 75 cells and a maximum of 250 cells per experimental group, according to the density of the cell culture. *** p < 0.01 vs. VEH (Student’s *t*-test).

To further characterize the morphological changes caused by cytostatic, non-lethal concentrations of mifepristone, the various cancer cell types were cultured in the presence of vehicle or mifepristone for 72 h, and the arrangement of filamentous actin (F-actin) and tubulin filaments contributing to cytoskeletal structure and overall cell morphology were assessed. Figure 5 depicts that mifepristone, in addition to causing changes in overall cell shape, disrupted the organization of both actin fibers and tubulin filaments. Confocal imaging revealed that untreated SKOV-3 cells possess cortical actin, stress fibers, and cell polarity as shown by the presence of lamellipodia (Figure 5A, left panel). Mifepristone caused a remarkable change in cell shape, loss of cortical actin and stress fibers, and gain of peripheral membrane ruffles rich in polymerized actin (Figure 5A, right panel). In U87MG cells, treatment with mifepristone did not change the distribution of cortical actin substantially, yet it increased the number of peripheral actin ruffles (Figure 5B). MDA-MB-231 breast cancer cells responded to mifepristone with a remarkable increase not only of peripheral actin ruffles, but also of circular dorsal actin ruffles or actin ribbons (Figure 5C). Finally in LNCaP cells, mifepristone, as in the other cancer cell types, caused an increase in the number of peripheral actin ruffles (Figure 5D). A commonality in all cancer cells under the effect of mifepristone was the increase in the number of membrane actin ruffles (Figure 5E). Tubulin, which in untreated and polarized cells usually arranges around the microtubule-organizing center and the Golgi apparatus [31], was mainly found framing the periphery of the nuclei in control cells; however, in mifepristone-treated cells, tubulin accumulated mainly in the long-thin neurite-like extensions (Figure 5). The complete microscopic fields of the cell cultures from which the images represented in Figure 5 were obtained, can be observed in Additional file 4: Figure S4.

To evaluate whether mifepristone was merely dysregulating the distribution of actin and tubulin or changing their abundance, we assessed the expression levels of one of the isotypes of actin, β-actin, and one of the isotypes of tubulin, α-tubulin. Given that actin and tubulin are both commonly used as loading controls in Western blot studies, we sought to evaluate any differences in their expression by loading lysates obtained from an equal number of vehicle or mifepristone-treated cells. Membranes were immunoblotted for β-actin, α-tubulin, and GAPDH, which was used as protein loading control. Densitometry analysis was performed and protein levels expressed as the ratio of β-Actin/GAPDH (Additional file 5: Figure S5A) or α-Tubulin/GAPDH (Additional file 5: Figure S5B). Mifepristone did not significantly change the expression levels of either β-actin or α-tubulin in any cell line, suggesting that the action of mifepristone is limited to dysregulating the distribution of the proteins and, consequently, the overall architecture of the cytoskeleton

### Effect of mifepristone on cellular de-adhesion and adhesion dynamics

**Figure 6:**
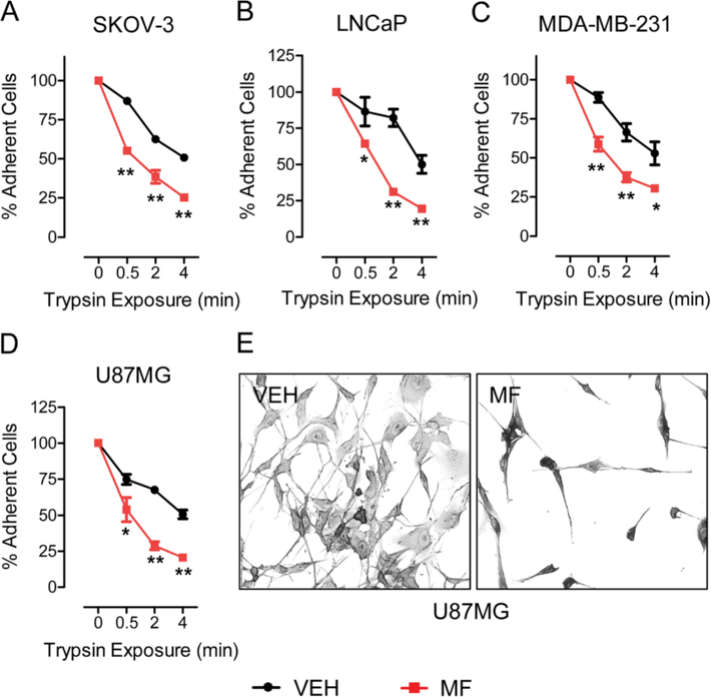
**Exposure to mifepristone impairs the ability of cancer cells to remain adherent.** SKOV-3 **(A)**, LNCaP **(B)**, MDA-MB-231 **(C)** or U87MG **(D)** cells were plated at equal densities and treatment with vehicle (VEH) or mifepristone (MF)-containing media was administered for 72 h. Cells were then exposed to 0.025% trypsin/0.265 mM EDTA for 0.5, 2, or 4 min. The detached cells were removed, while those remaining adherent were fixed with 100% methanol and stained with 0.25% crystal violet (as shown in panel **E** for U87MG cells; 4 min trypsin exposure; 200 X). Stained cells were then imaged and counted via bright field microscopy. Adherent cells were quantified as a percent of control (0 min trypsin exposure). Data shown represent the mean ± s.e.m. of 3 independent experiments completed in triplicate. * p < 0.01 vs. VEH; ** p < 0.001 vs. VEH (**A-D**). Statistical analysis was done using two-way ANOVA followed by Bonferroni’s post-tests.

One commonality in the cancer cells that were treated with cytostatic doses of mifepristone was the increased density of membrane actin ruffles along the surface of the cells (Figure 5). Actin ruffles are sheet-like membrane protrusions that do not adhere to the substratum and increase in number whenever the adhesion of a cell to the substratum is not optimal [32,33]. Consequently, we first investigated whether cells that are already attached, once treated with mifepristone, are loosely adhered and, secondly, whether pre-treatment with mifepristone affected the adhesion capacity of cells to extracellular matrix-coated surfaces. To answer the first question, we assessed the capability of cells to remain attached under treatment with mifepristone via a cell de-adhesion assay. The cells were plated at equal densities and treated with mifepristone for 72 h, at which point they were exposed to a very low concentration of trypsin/EDTA for short periods of time; these conditions do not allow for the optimal detachment of cells that are well adhered. All cells that detached from the plate were removed, and those remaining were fixed, stained, and counted. In all cases, cells pre-treated with mifepristone, having had they morphology altered, detached at a significantly faster rate than those untreated. This effect was seen as early as 30 sec following induction of de-adhesion in all cell lines (Figure 6A-D). Figure 6E shows a representative field of U87MG cells remaining in the plate after 4 min of induction of de-adhesion. Mifepristone-pretreated cells, which are scarce in the culture field, still show their thin and elongated neurite-like extensions, a consequence of mifepristone action (Figure 6E, right panel). In contrast, vehicle-treated cells exposed to the same de-adhesion conditions as their mifepristone-treated counterparts, were less sensitive to the mild trypsinization procedure, and depict normal morphology (Figure 6E, left panel).

**Figure 7:**
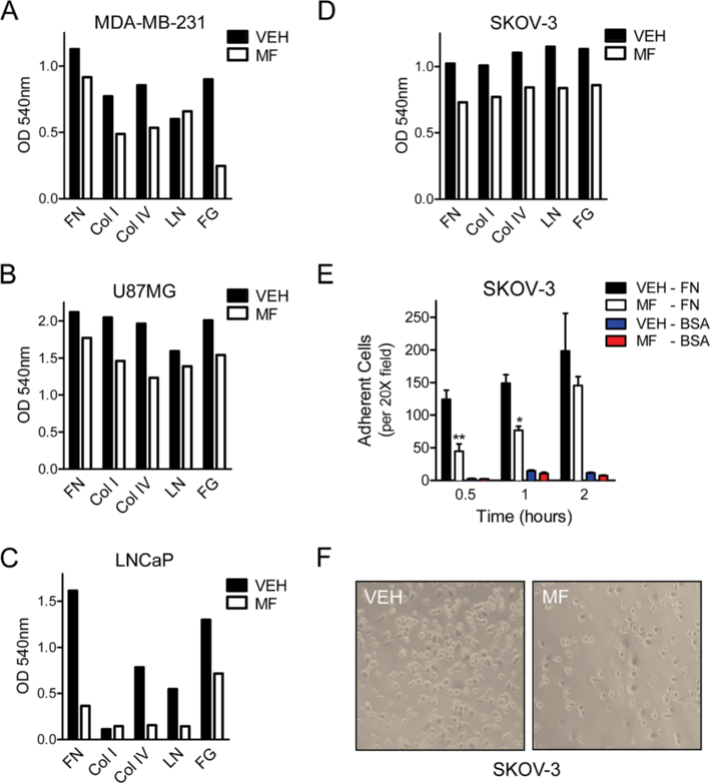
**Mifepristone-pretreated MDA-MB-231 (A), U87MG (B), LNCaP (C), and SKOV-3 (D) cells have delayed adhesion to extracellular matrix proteins.** For all cell lines, treatment with cytostatic concentrations of mifepristone was administered for 72 h prior to plating. After treatment, cells were trypsinized and re-plated in a pre-coated plate at a density of 100,000 cells per well. FN: fibronectin, Col I: collagen I, Col IV: collagen IV, LN: Laminin, FG: Fibrinogen. Data shown in **A-D** represent one experiment that was repeated three times with a similar outcome. In **E**, SKOV-3 cells were cultured in the presence of vehicle (VEH) or mifepristone (MF) for 72 h. The cells were trypsinized and placed in plates that had been pre-coated with 0.1% fibronectin or bovine serum albumin (BSA) used as a negative control of adhesion. Adhesion was quantified as the number of cells attached per 20 X microscopic field. * p < 0.05 vs. VEH; ** p < 0.01 vs. VEH. Statistical analysis was done using two-way ANOVA followed by Bonferroni’s post-test. Bright field Images in **F** are representative of vehicle (VEH) or mifepristone (MF)-pretreated cells observed after 60 min of incubation (200 X).

To assess whether mifepristone impairs the capacity of cancer cells to adhere to extracellular matrix, cells were pre-treated for 72 h with or without a cytostatic concentration of mifepristone, trypsinized, and re-plated in commercially available plates that had been pre-coated with an array of extracellular matrix proteins including fibronectin, collagen I, collagen IV, laminin or fibrinogen. Cells were allowed 1 h to adhere, except for LNCaP cells that underwent adhesion for 24 h. Thereafter, the cells were stained and optical density (OD) was measured. All cell lines showed different kinetics of adhesion to the substrates offered, as observed by the different ranges of OD detected (Figure 7A-D). Mifepristone pre-treated cells, in all cell lines studied, had diminished adhesion to each one of the surfaces within a particular time-frame (Figure 7A-D). Data presented in Figure 7 represents one experiment that was repeated three times with a similar trend, yet we found variability in the overall adhesion capacity of the cell preparations from one experiment to the next. Consequently we semi-quantitated the data from three experiments; we defined a strong inhibitory effect of mifepristone when there was a decrease in OD reading of more than 50% as compared to vehicle treated cells; similarly, a slight inhibitory effect was defined as a 10-50% of OD decrease. Finally, a decrease in OD reading of less than 10% was considered as no effect. In all cell lines, either a slight or a strong inhibitory effect of mifepristone on cell adhesion was observed along the three independent experiments (Additional file 6: Table S1).

To confirm the impairment of mifepristone-pretreated cells to adhere to an extracellular matrix-related protein, an equal number of either vehicle-pretreated or mifepristone-pre-treated SKOV-3 cells were incubated at 37°C for various times (0.5, 1, or 2 h) on plates that had been pre-coated with fibronectin. Thereafter, the cultures were washed to remove the non-adherent cells, whereas the cells that had already attached to the plate at each time point were fixed with methanol, stained with crystal violet, and their number was quantified per microscopic field. The results shown in Figure 7E clearly indicate that pretreatment with mifepristone significantly delayed the adherence of SKOV-3 cells to fibronectin. Figure 7F depicts representative bright field images of vehicle- or mifepristone-pretreated SKOV-3 cells that had attached to a fibronectin-coated surface for 60 min; it can be clearly observed the reduced number of adhered cells that had been pre-treated with mifepristone (Figure 7, right panel) when compared to those exposed only to vehicle (Figure 7, left panel).

## Discussion

In this study we observed that, at known cytostatic doses, mifepristone induced a strikingly similar change in shape of four highly metastatic and aggressive cancer cell lines. This included shrinkage of the cell body with long, thin, neurite-like cellular extensions. Morphology changes were found to be dependent on time of exposure to the drug, with confirmed and observable phenotypic changes occurring after 48 h. These alterations were not influenced by the cellular density of culture, suggesting that mifepristone has an intrinsic effect upon cellular structure, and that this change in morphology is not a result of lack of perceived cell number. In addition, treatment with mifepristone was not associated with changes in total expression of β-actin and α-tubulin.

Mifepristone is known to exert anti-progestin activity by blocking the action of progesterone on progesterone receptors (PR) [34]. In cancer cells expressing high levels of PR, nanomolar concentrations of mifepristone have been shown to be sufficient to block cell growth ([8] and references cited therein). In the present work, in cell lines not expressing canonical PR [26], we used concentrations of mifepristone in the micromolar range, higher than those required to bind PR, suggesting that a different mechanism not involving classical PR is at work. Such mechanism, which still needs to be unveiled, may be relevant to target with mifepristone cancers not expressing standard PR.

Potential mediators of the effect of mifepristone are the glucocorticoid receptors (GR). It was reported that mifepristone binds GR when used at concentrations higher than those needed to bind PR [35]. We previously addressed that the only commonality among the four cell lines studied in the present work was the expression of the beta isoform of the GR (GR-β) [26]. This is of interest as GR-α is considered the driver of glucocorticoid effects upon regulation of gene transactivation, whereas GR-β has been mostly considered a dominant negative isoform [36]. There are however reports indicating that in cells forced to express only GR-β, mifepristone was the only one out of more than 50 steroids capable of binding the receptor [37] and of regulating the activity of a reporter gene [38].

When operating at micromolar concentrations, mifepristone has a distinctive effect not shared by other natural and synthetic steroids. For instance, when used at equimolar concentrations, mifepristone was a much potent inhibitor of ovarian cancer cell growth than progesterone, medroxyprogesterone acetate or levonorgestrel [17]. Furthermore, equimolar concentrations of the GR agonist dexamethasone did not inhibit growth when compared to mifepristone or to two other related antiprogestins, ORG31710 and CDB2914 [39]. At the concentration utilized, dexamethasone was able to down regulate the expression of GR-α and GR-β isoforms, whereas the antiprogestins did not, suggesting that they have different mechanisms of action despite reaching the cells at micromolar levels [39].

Important in studying mifepristone as a possible treatment option in oncology was to address its long-term effect on the cancer cells. Interestingly, upon removal of mifepristone following exposure for 3 days, cell morphology and proliferation returned to that of untreated cells in all cell lines except for the LNCaP prostate cancer cells. In these cells, the non-proliferative effect of mifepristone remained as long as 9 days following treatment removal, suggesting an irreversible growth arrest. Previous research has shown the propensity of LNCaP cells to enter irreversible growth arrest and senescence in response, for instance, to treatment with doxorubicin or to culture in androgen-free media [29,40]. We confirmed that LNCaP cells exposed to mifepristone become senescent upon removal of the drug as indicated by the increase in the activity of perinuclear SA-β-galactosidase. One possible explanation as to why, from a panel of 4 cells lines, only LNCaP cells underwent senescence following mifepristone, relies in the likely induction of the tumor suppressor gene p16^Ink4A^. While SKOV-3 and U87MG cell lines are null for p16^Ink4A^[41-43], and MDA-MB-231 has a homozygous deletion of p16^Ink4A^[44,45], LNCaP cells retain the p16^Ink4A^ gene [46]. Cells that undergo senescence have been reported to upregulate p16^Ink4A^[47-49], and not to regrow in response to overexpression of oncogenic Ras [50]. The phenomenon of senescence has been studied both *in vitro* and *in vivo*, and pro-senescence therapy has rapidly become a target for cancer treatment [51]. Whereas it was shown that senescence occurs naturally in benign tumors of melanocytes [52], it was also found that cellular senescence can be induced *in vivo* and block, for example, prostate tumorigenesis [53]. Also, the use of chemotherapy to induce senescence has been shown to be successful in mice models, leading to an anti-tumor effect with a corresponding increase in p16^Ink4A^[54]. The ability of mifepristone to induce senescence in p16^Ink4A^-positive prostate cancer cells provides yet another rationale for its potential use as an anti-cancer agent, in particular in cells carrying wild type versions of the p16^Ink4A^ tumor suppressor gene.

An alternative explanation for the senescence induced by mifepristone in LNCaP cells is the possible mediation by androgen receptors (ARs). LNCaP is the only cell line in the studied cohort that expresses ARs [26], which have been found able to bind mifepristone [55]. Thus, the role of both p16 ^Ink4A^ as well as ARs in the mediation of mifepristone-induced senescence in LNCaP cells deserves to be investigated.

Mifepristone likely altered cellular morphology as a consequence of the dysregulation of the cytoskeletal structure, which was observed via fluorescent staining of actin fibers (F-actin) and tubulin filaments; these were both found to rearrange in response to mifepristone. Actin fibers were found reorganized to sites located at the ends of tubulin-rich extensions. As this was seen multiple times in different directions within individual cells, there appears to be a loss of cell polarity following mifepristone treatment. Upon mifepristone treatment, tubulin filaments were mainly located in the neurite-like extensions, in contrast to their original localization throughout the cell body with particular intensity around the nucleus observed in controls.

F-actin is formed by polymerization of actin molecules that assemble at different times and locations, depending upon the extracellular environment [32]. These actin-based structures interact with one another or with microtubule-based structures, reflecting the complexity of the dynamics of the cytoskeleton [56]. Among the actin-based structures are: i) cortical actin, which mostly defines the shape of the cell; ii) finger-like protrusions termed filopodia that are adhered in some manner to a substratum or another cell, and are believed to function as directional sensors; iii) stress fibers that are contractile actomyosin bundles essential for cell adhesion to the substratum and for changes in cell morphology during migration; iv) lamellipodia, which are surface-attached sheet-like, membrane protrusions with weak adherence, and observed during cell motility and spreading; and v) ruffles, which are sheet-like membrane protrusions or flat membrane folds from the cortical cytoskeleton that do not attach whatsoever to the extracellular matrix. Ruffles are formed as a consequence of inefficient integrin-ligand interaction at the leading edge of lamellipodia and contain densely packed arrays of thin actin filaments [33]. A high frequency of ruffle formation is usually associated with low lamellipodia formation and inefficient cell adhesion and migration [33]. We propose that mifepristone induces the accumulation of membrane ruffles and a reduction in lamellipodia, thus destabilizing the formation of cell substrate adhesions by integrins that connect the cytoskeleton with the extracellular matrix [57]. Under mifepristone treatment, the formation of adhesions may be inefficient because lamellipodia may not have the appropriate anchorage, becoming detached and retracted toward the main cell body, thus forming membrane ruffles. The behavior of mifepristone-treated cells supports the data of Born et al. [33], suggesting that high ruffling rates are indicative of inadequate adhesion, whereas low ruffling rates are associated with optimal adhesion. While the majority of nascent adhesions undergo rapid turnover such that their components can be incorporated into newly formed adhesion sites, a few mature behind the leading edge in response to tensile stress and increase in size [58]. Adhesion turnover may be blocked by mifepristone leading to the accumulation of actin ruffles that mature and do not adhere to the substratum. We suggest that the more ruffles, the less surface area mifepristone-treated cells would have to actually develop the needed focal adhesion complexes to link the cytoskeleton, the integrins and the extracellular matrix.

We observed that cells under the stress of mifepristone are easily de-adhered from the extracellular surface than untreated cells when exposed to a sub-optimal concentration of trypsin. Because an inverse relationship between de-adhesion time and cell contractility assessed by trypsin-induced de-adhesion has been demonstrated [59], by altering cytoskeletal dynamics, mifepristone may interfere with the molecular link between the actin cytoskeleton and the extracellular matrix. In addition, owing to the fact that changes in shape and cytoskeletal remodeling are coupled to the cell cycle machinery governing the G1/S transition [60-62], it is possible that the cell growth inhibition caused by mifepristone, which we previously demonstrated to be associated with G1-S cell cycle arrest, blockage of cyclin dependent kinase 2 activity, and accumulation of cyclin dependent kinase inhibitors p27^kip1^ and p21^cip1^[14,17,26], may be secondary to a primary effect on the cytoskeleton.

Since the survival, movement and invasiveness of cancer cells in the organism require great plasticity in the distribution of F-actin, our data suggest the mifepristone may interfere with such actin polymerization dynamics, disturbing the metastatic process. Blocking actin plasticity with mifepristone can be therapeutically beneficial to reduce the seeding at secondary sites by cells that had detached from a primary tumor.

Microtubules are important to maintain cell shape, play a key role in the polarized distribution of signals within a cell, and have been implicated in the asymmetric regulation of adhesion dynamics; in particular, they promote adhesion disassembly triggering the destabilization first, and then the detachment of adhesion components. At the same time, adhesions can be pulled off the substrate by stress fibers, which contract in response to microtubule depolymerization [27]. We observed a lack of radial distribution of tubulin filaments from the center of the cells in response to mifepristone; instead we visualized an increase of tubulin fibers located in the neurite-like extensions of the cells where the membrane actin ruffles became abundant, suggesting a connection between redistribution of microtubules and dysregulated adhesion. Usually an intact microtubule network with dynamic properties that are asymmetric has the full potential to coordinate adhesion dynamics in different regions of the cells, allowing directional migration [27]. Mifepristone may disrupt this dynamic equilibrium, blocking adhesion dynamics, and, tentatively, migration as well. Further studies are necessary to elucidate the relationship between mifepristone treatment, membrane ruffling, tubulin rearrangement, and cellular adhesion.

## Conclusions

The anti-cancer effect of mifepristone manifested in the inhibition of cellular growth is related to drastic alternations in cellular morphology with the formation of neurite-like protrusions and altered cytoskeletal architecture characterized by an increase in membrane F-actin ruffling and concentration of tubulin filaments at the neurite-like cellular extensions. Such effect of mifepristone is associated with dysregulated cellular adhesion; it is reversible in most cell lines, except for prostate cancer cells that instead undergo senescence. Whether or not mifepristone-induced cytostasis, and alterations in cell shape, cytoskeletal structure, and adhesion capacity affect the migratory and invasive properties of cancer cells, warrant further investigations.

## Competing interests

The authors declare that there is no conflict of interest that could influence the impartiality of the research reported.

## Authors’ contributions

Conceived and designed experiments: BNB AAG CMT. Performed experiments BNB CRT TMU MST AAG. Analyzed the data: BNB AAG CMT. Contributed reagents/materials/analysis tools: CMT. Wrote the paper: BNB CMT. All authors read and approved the final manuscript.

## Pre-publication history

The pre-publication history for this paper can be accessed here:

http://www.biomedcentral.com/1471-2407/13/35/prepub

## Supplementary Material

Additional file 1: Figure S1 Time-course effect of mifepristone on cancer cells of the ovary (A), breast (B), prostate (C), and nervous system (D). Cells were seeded at a density appropriate for exponential growth for each cell line, allowed to adhere for 24 h, and then exposed to a previously determined cytostatic concentration of mifepristone (MF) for 72 h. Cells that received vehicle for 72 h were used as positive control of growth (right panel, VEH). Images were taken using phase contrast microscopy every 12 h and examined for morphological changes. Scale bar = 50 μm. Click here for file

Additional file 2: Figure S2 Treatment of SKOV-3 cells with mifepristone induced inhibition of growth associated with changes in cell morphology. Cells were cultured in 8-well chamber slides in the presence of vehicle (VEH) or 20 μM mifepristone (MF) for 4 days. At the end of the incubation cells were fixed with 4% paraformaldehyde and stained with hematoxylin. X 400. Click here for file

Additional file 3: Figure S3 Long-term effect of mifepristone on LNCaP cells. Cells were seeded, allowed to adhere for 24 h, and then exposed to a cytostatic concentration of mifepristone (MF) for 72 h. Thereafter, triplicate wells were harvested by trypsinization and counted by microcytometry. Remaining wells were returned to vehicle treatment and monitored after 1, 3, 7, or 9 days for their growth in the absence of MF and compared against the growth of similar number of cells never exposed to the steroid. Click here for file

Additional file 4: Figure S4 Effect of mifepristone on the cellular distribution of filamentous actin (F-actin) and tubulin. SKOV-3 cells, U87MG, MDA-MB-231 and LNCaP were cultured in the presence of vehicle (VEH) or mifepristone (MF) for 72 h, following which immunocytochemistry was used to visualize the cytoskeletal protein α-tubulin, AlexaFluor 594^®^ phalloidin was used to visualize filamentous actin (F-actin), and DAPI to label cell nuclei. Images were taken using confocal microscopy. Scale bar = 50 μm. Click here for file

Additional file 5: Figure S5 Expression of β-actin and α-tubulin in mifepristone-treated cells. Cells were plated and exposed to either vehicle (VEH) or the cytostatic concentration of mifepristone (MF) optimized previously for each cell line for 72 h. Following treatment, cells were subsequently harvested, lysed, and whole-protein extracts, representing equal numbers of VEH or MF-treated cells were separated by electrophoresis. Immunoblots were then probed for β-actin and α-tubulin. GAPDH was included as a loading control. Densitometry analysis was performed from three different experiments and protein levels expressed as the ratio of β-actin/GAPDH (A), or α-tubulin/GAPDH (B). Densitometry graphs represent the mean ± s.e.m. Click here for file

Additional file 6: Table S1 Semi-quantitative representation of the effect of mifepristone on the adhesion of cells to individual extracellular matrix proteins. Click here for file
