# Supplementary figures and images for: Cytostasis and morphological changes induced by mifepristone in human metastatic cancer cells involve cytoskeletal filamentous actin reorganization and impairment of cell adhesion dynamics
Source: BMC Cancer. 2013 Jan 26;13:35. doi: 10.1186/1471-2407-13-35 (PMC3562154; doi:10.1186/1471-2407-13-35)

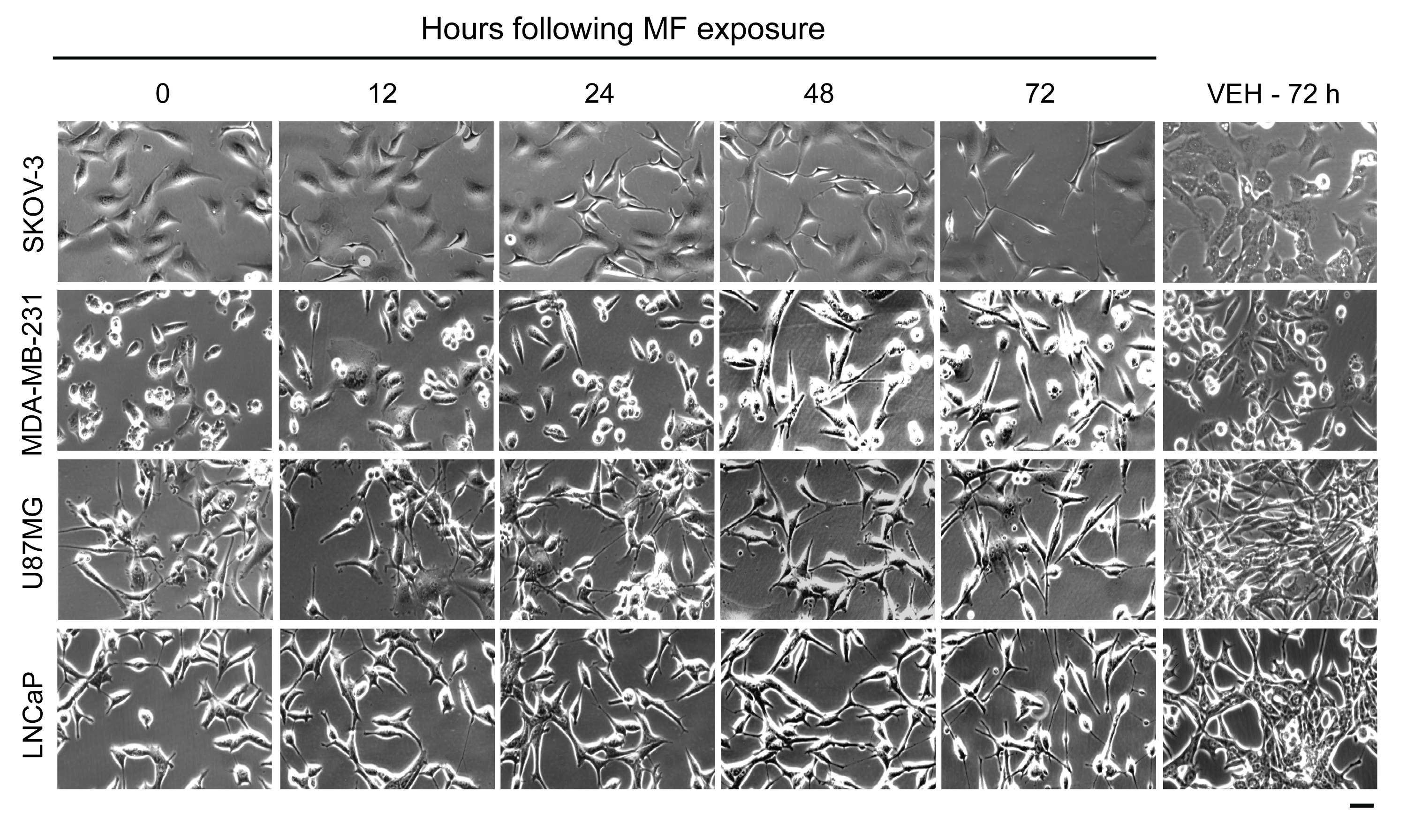

Supplement: Additional file 1: Figure S1 — Time-course effect of mifepristone on cancer cells of the ovary (A), breast (B), prostate (C), and nervous system (D). Cells were seeded at a density appropriate for exponential growth for each cell line, allowed to adhere for 24 h, and then exposed to a previously determined cytostatic concentration of mifepristone (MF) for 72 h. Cells that received vehicle for 72 h were used as positive control of growth (right panel, VEH). Images were taken using phase contrast microscopy every 12 h and examined for morphological changes. Scale bar = 50 μm. [file 1471-2407-13-35-S1.tiff]

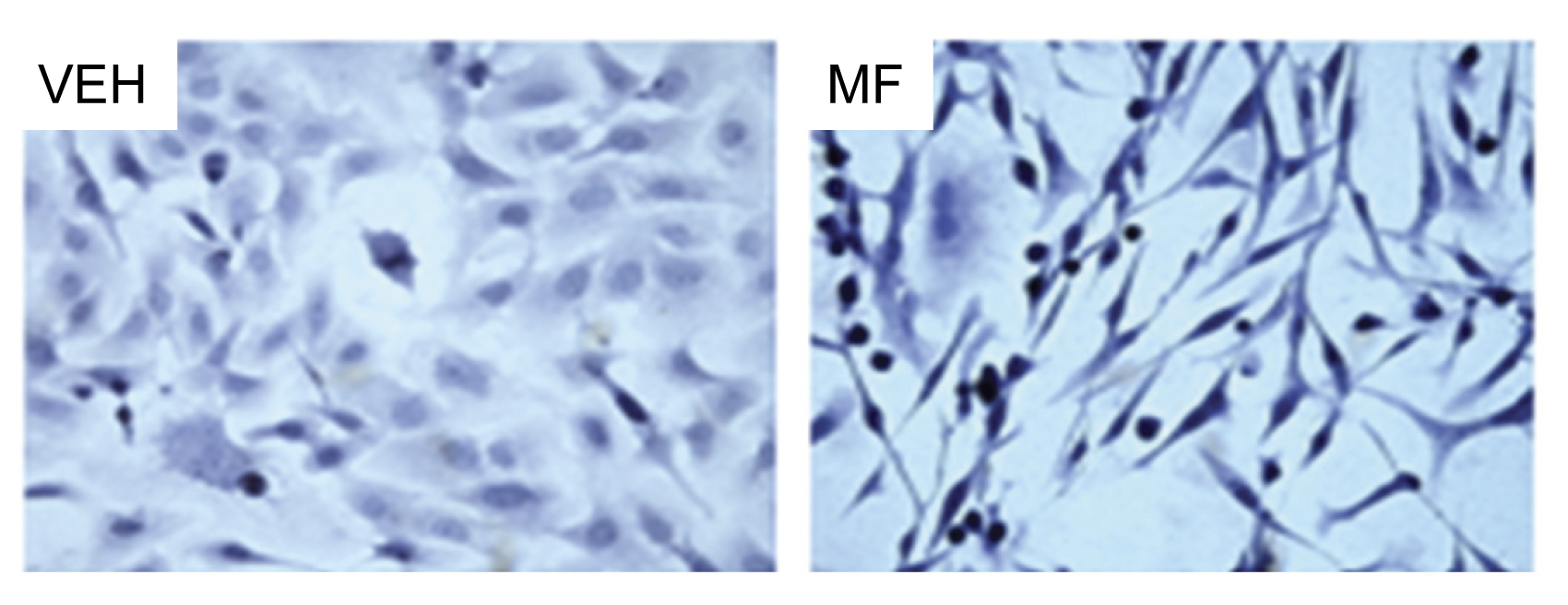

Supplement: Additional file 2: Figure S2 — Treatment of SKOV-3 cells with mifepristone induced inhibition of growth associated with changes in cell morphology. Cells were cultured in 8-well chamber slides in the presence of vehicle (VEH) or 20 μM mifepristone (MF) for 4 days. At the end of the incubation cells were fixed with 4% paraformaldehyde and stained with hematoxylin. X 400. [file 1471-2407-13-35-S2.tiff]

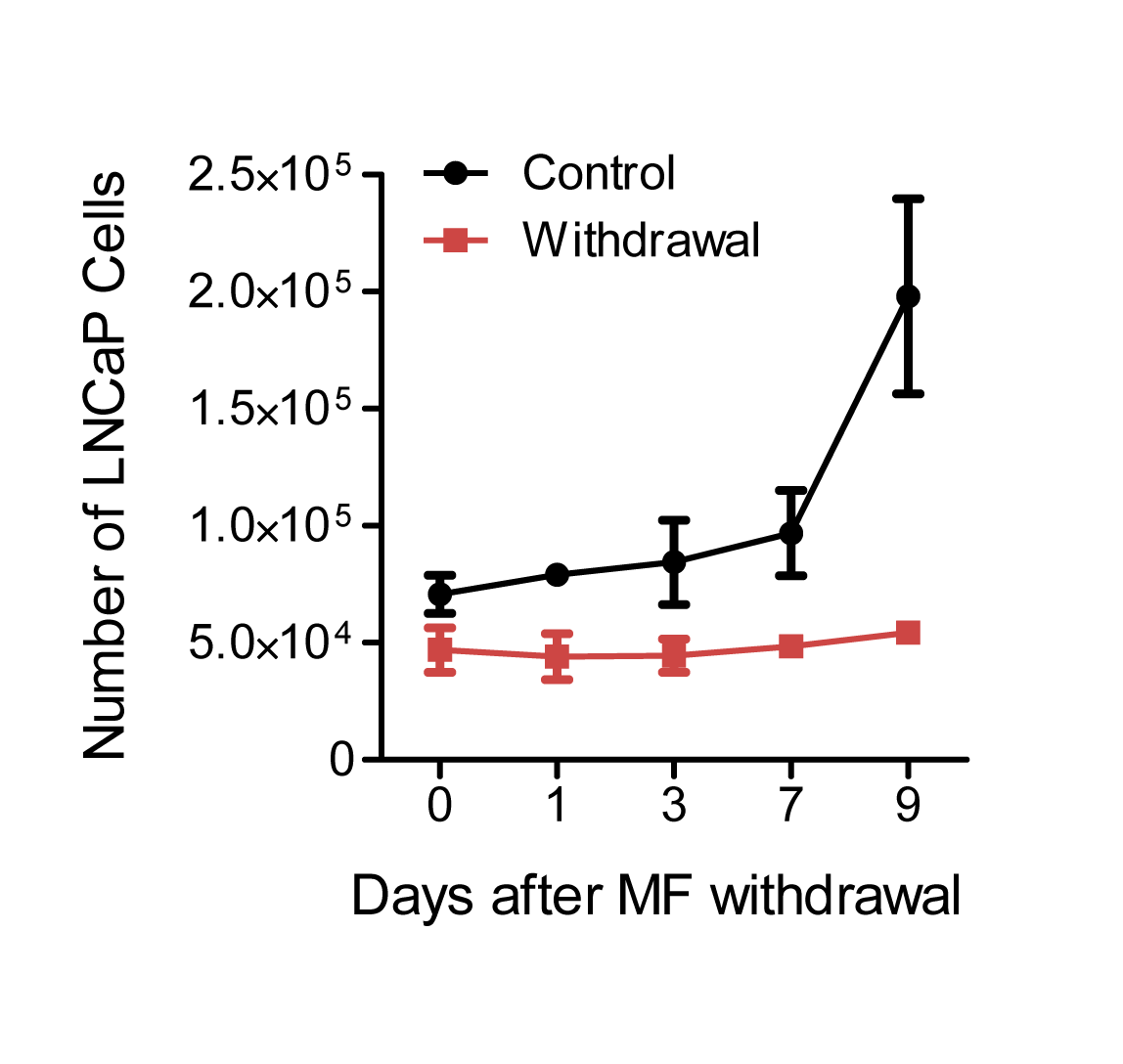

Supplement: Additional file 3: Figure S3 — Long-term effect of mifepristone on LNCaP cells. Cells were seeded, allowed to adhere for 24 h, and then exposed to a cytostatic concentration of mifepristone (MF) for 72 h. Thereafter, triplicate wells were harvested by trypsinization and counted by microcytometry. Remaining wells were returned to vehicle treatment and monitored after 1, 3, 7, or 9 days for their growth in the absence of MF and compared against the growth of similar number of cells never exposed to the steroid. [file 1471-2407-13-35-S3.tiff]

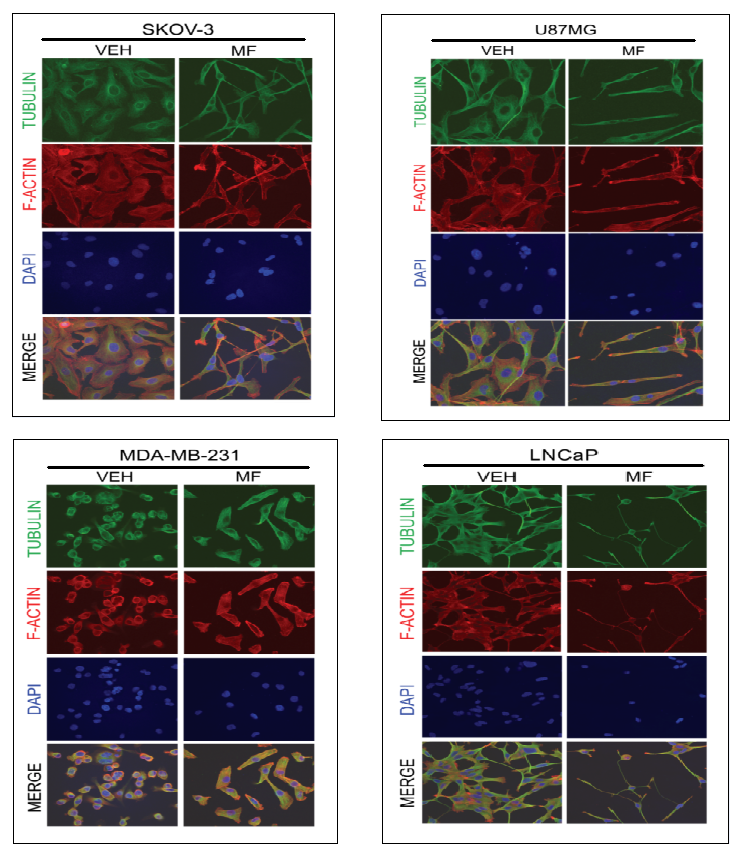

Supplement: Additional file 4: Figure S4 — Effect of mifepristone on the cellular distribution of filamentous actin (F-actin) and tubulin. SKOV-3 cells, U87MG, MDA-MB-231 and LNCaP were cultured in the presence of vehicle (VEH) or mifepristone (MF) for 72 h, following which immunocytochemistry was used to visualize the cytoskeletal protein α-tubulin, AlexaFluor 594® phalloidin was used to visualize filamentous actin (F-actin), and DAPI to label cell nuclei. Images were taken using confocal microscopy. Scale bar = 50 μm. [file 1471-2407-13-35-S4.tiff]

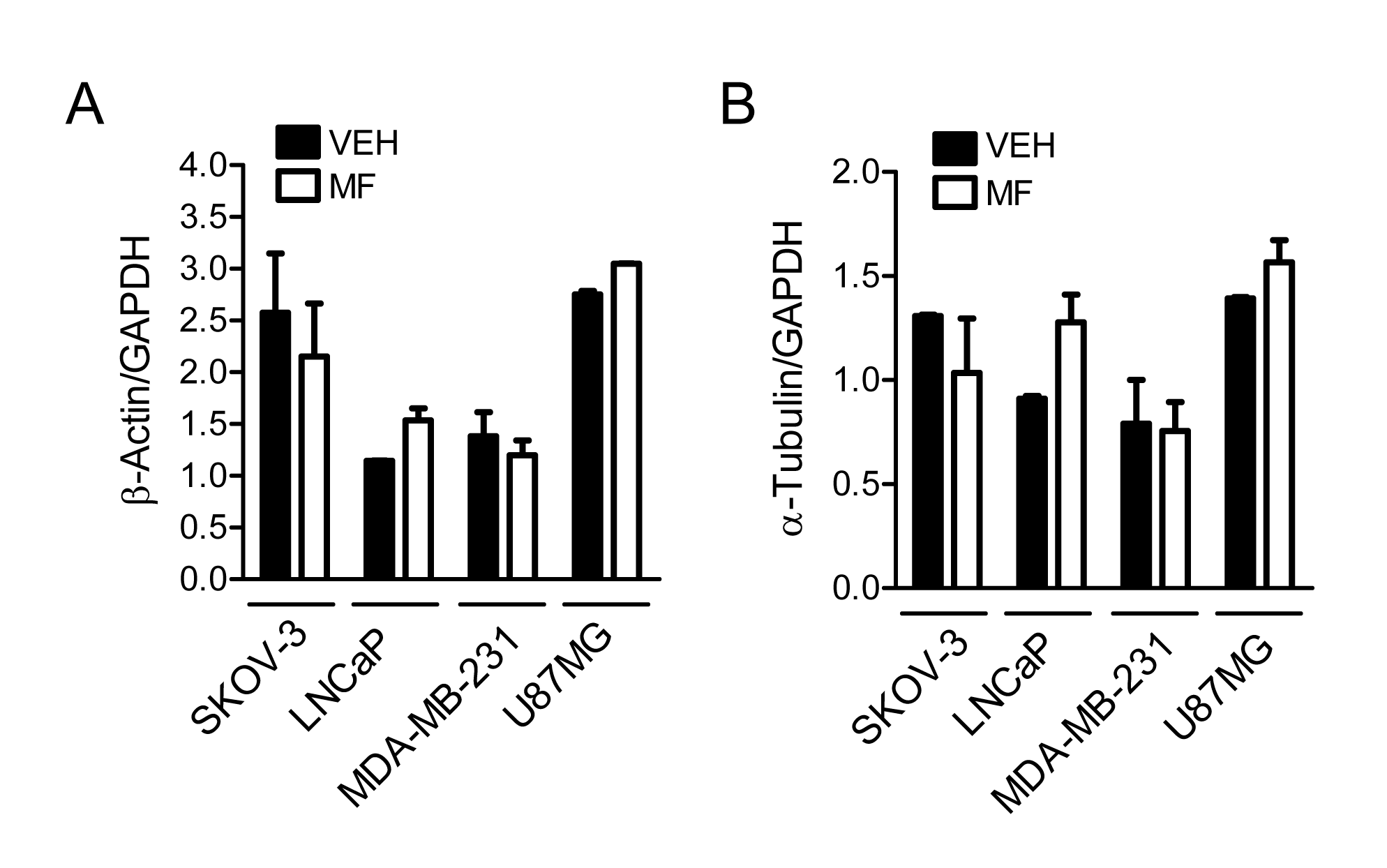

Supplement: Additional file 5: Figure S5 — Expression of β-actin and α-tubulin in mifepristone-treated cells. Cells were plated and exposed to either vehicle (VEH) or the cytostatic concentration of mifepristone (MF) optimized previously for each cell line for 72 h. Following treatment, cells were subsequently harvested, lysed, and whole-protein extracts, representing equal numbers of VEH or MF-treated cells were separated by electrophoresis. Immunoblots were then probed for β-actin and α-tubulin. GAPDH was included as a loading control. Densitometry analysis was performed from three different experiments and protein levels expressed as the ratio of β-actin/GAPDH (A), or α-tubulin/GAPDH (B). Densitometry graphs represent the mean ± s.e.m. [file 1471-2407-13-35-S5.tiff]
