# Supplementary material for: Cytostasis and morphological changes induced by mifepristone in human metastatic cancer cells involve cytoskeletal filamentous actin reorganization and impairment of cell adhesion dynamics
Source: BMC Cancer. 2013 Jan 26;13:35. doi: 10.1186/1471-2407-13-35 (PMC3562154; doi:10.1186/1471-2407-13-35)
Supplement: Additional file 6: Table S1 — Semi-quantitative representation of the effect of mifepristone on the adhesion of cells to individual extracellular matrix proteins. [file 1471-2407-13-35-S6.doc]

**Additional File 9: Table S1. Semi-quantitative representation of the effect of mifepristone on the adhesion of cells to individual extracellular matrix proteins**

|  | **SKOV-3** | **LNCaP** | **MDA-MB-231** | **U87MG** |
| --- | --- | --- | --- | --- |
| Fibronectin | **↓↓** | **↓↓** | **↓** | **↓** |
| Collagen I | **↓↓** | **↓** | **↓↓** | **↓** |
| Collagen IV | **↓** | **↓** | **↓** | **↓** |
| Laminin | **↓** | **↓** | **↓** | **↓** |
| Fibrinogen | **↓** | **↓** | **↓↓** | **↓** |

Adhesion assays, using a pre-coated plate, were performed in triplicate. Optical density (OD) at 540 nm was obtained and compared semi-quantitatively between all assays. A decrease in measured OD of mifepristone pre-treated versus untreated cells of less than 10% was considered as no effect; between 10-50% was considered slightly inhibitory (one arrow); more than 50% was considered strongly inhibitory (two arrows).
